# Maculopathy Masquerading as Migraine

**DOI:** 10.3390/vision5030040

**Published:** 2021-08-27

**Authors:** Laura C. E. Drayer Turner, Jan A. Coebergh, Philip J. Banerjee

**Affiliations:** 1 Frimley Health NHS Foundation Trust, Portsmouth Road, Frimley, Camberley GU16 7UJ, UK; ldrayerturner@nhs.net; 2 Sussex Eye Hospital, University Hospitals Sussex NHS Foundation Trust, Eastern Road, Brighton BN2 5BF, UK; 3 Ashford St Peter’s NHS Foundation Trust, Guildford Street, Lyne, Chertsey KT16 0PZ, UK; jan.coebergh@nhs.net

**Keywords:** scotomata, migraine, maculopathy, acute macular neuroretinopathy, persistent negative aura

## Abstract

We describe a case of a 23-year-old Caucasian woman with a background history of migraines who presented with bilateral paracentral scotomata. The ophthalmoscopy and MRI head were originally thought to be normal, and the scotomata were attributed to be of migrainous origin: a persistent negative aura. However, persistence of her symptoms prompted further specialist review 10 months later, at which time subtle bilateral perifoveal changes were noted, which had been apparent but overlooked at the initial assessment. Near-infrared reflectance imaging enabled better visualization of the lesions, which were apparent prior to any abnormalities on clinical examination. Spectral-domain optical coherence tomography revealed the early findings of hyperreflectivity in the outer nuclear and outer plexiform layers characteristic of acute macular neuroretinopathy. Our case aims to emphasize the importance of scrutinising ancillary tests of the macula in patients presenting with scotomata or atypical migraine symptoms, and to caution clinicians against diagnosing migraine with persistent negative aura without these investigations.

## 1. Introduction

Acute macular neuroretinopathy (AMN) is a rarely encountered condition first described by Bos and Deutman in 1975 in healthy young women taking oral contraceptive medication, who reported acute paracentral scotomata [1]. Whilst these symptoms are not specific, scotomata being seen in many other ophthalmic and neurological conditions, including as a form of visual aura in migraine, findings on clinical examination and ancillary tests may help diagnose them. However, the characteristic circumscribed intraretinal wedge-shaped red-brown lesions of AMN found in the macula and pointing towards the fovea, may initially be clinically undetectable [2]. The advent of an increasing repertoire of ancillary testing has helped address this diagnostic challenge: the lesions are better visualised on near-infrared reflectance imaging, and optical coherence tomography (OCT) analysis has helped localise the primary site of the abnormalities to the outer retina [3]. Initial features on OCT include hyperreflectivity of the outer nuclear and outer plexiform layers, with findings evolving over the course of the condition to outer retinal thinning [4].

Since the original description of the condition, an increasing range of underlying associations has been recognised, including preceding non-specific febrile illness, systemic shock, exposure to sympathomimetic agents (stimulant drugs that mimic the effects of adrenaline), and non-ocular trauma [2]. The pathophysiology of the condition, however, remains elusive. An ischaemic insult to the outer retinal and choroidal circulation has been postulated [5,6], a mechanism that may account for the vasoactive nature of some of its associations.

We describe a case of acute macular neuroretinopathy in a young female patient whose symptoms were attributed to migraine for a long period. The patient has given consent for the publication of this report.

## 2. Case Presentation

A 23-year-old Caucasian female was seen in her local Emergency Eye Clinic in February 2020 having suddenly noticed missing patches in her paracentral vision in both eyes—three in the right eye and one in the left—initially in the context of some intermittent frontal headaches. There was history of preceding coryzal (common cold-like) symptoms prior to the onset, but without additional associated symptoms. She was otherwise well. She had no previous ocular history, but had a background of migraines. She described her migraine episodes as classic visual aura followed by nausea and vomiting, with a subsequent severe frontal headache that was usually self-limiting over 48 to 72 h. Her migraine frequency and intensity had peaked in her adolescence but had continued to occur annually, thereafter. The last episode was approximately 8 months prior to her presentation. She was not on any regular medication at this time. There was no medical or ophthalmic family history of note.

**Figure 1:**
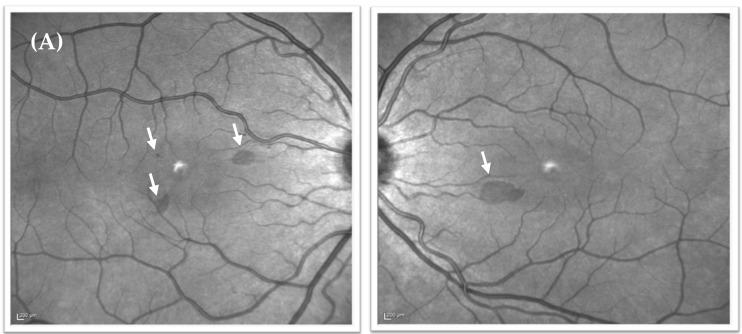

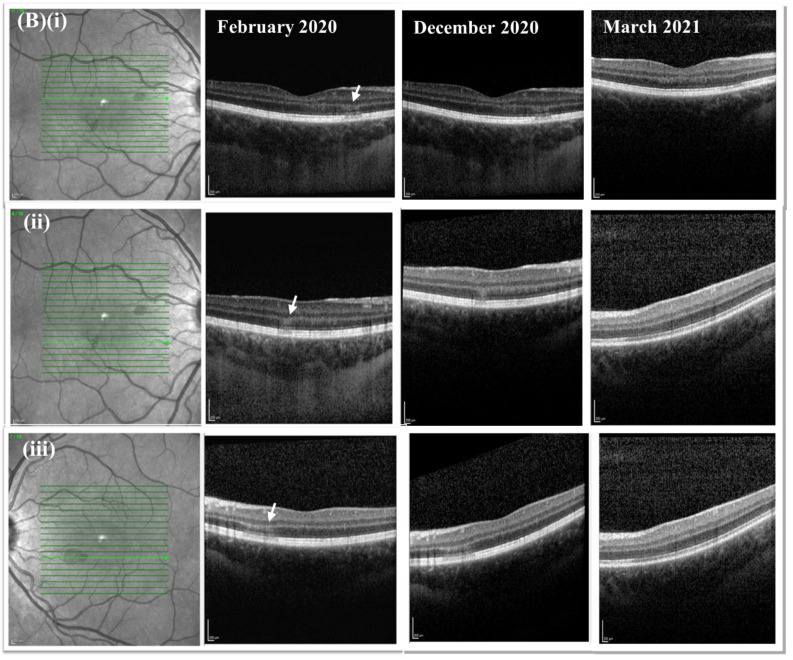
Near-infrared reflectance images from February 2020, and SD-OCT images from February 2020, December 2020, and March 2021. (**A**) Near-infrared reflectance images taken at presentation (February 2020) showing three discrete hyporeflective perifoveal lesions in the right eye and one in the left eye (white arrows). (**B**) Sequence over time of corresponding SD-OCT images through the two larger lesions in the right eye (**i**,**ii**) and the lesion in the left eye (**iii**), initially showing hyperreflectivity in the ONL and OPL with loss of clarity in the outer retinal bands (white arrows). This hyperreflectivity is seen to resolve over a 13-month period accompanied by improvement in the clarity of the outer retinal band architecture.

**Figure 2:**
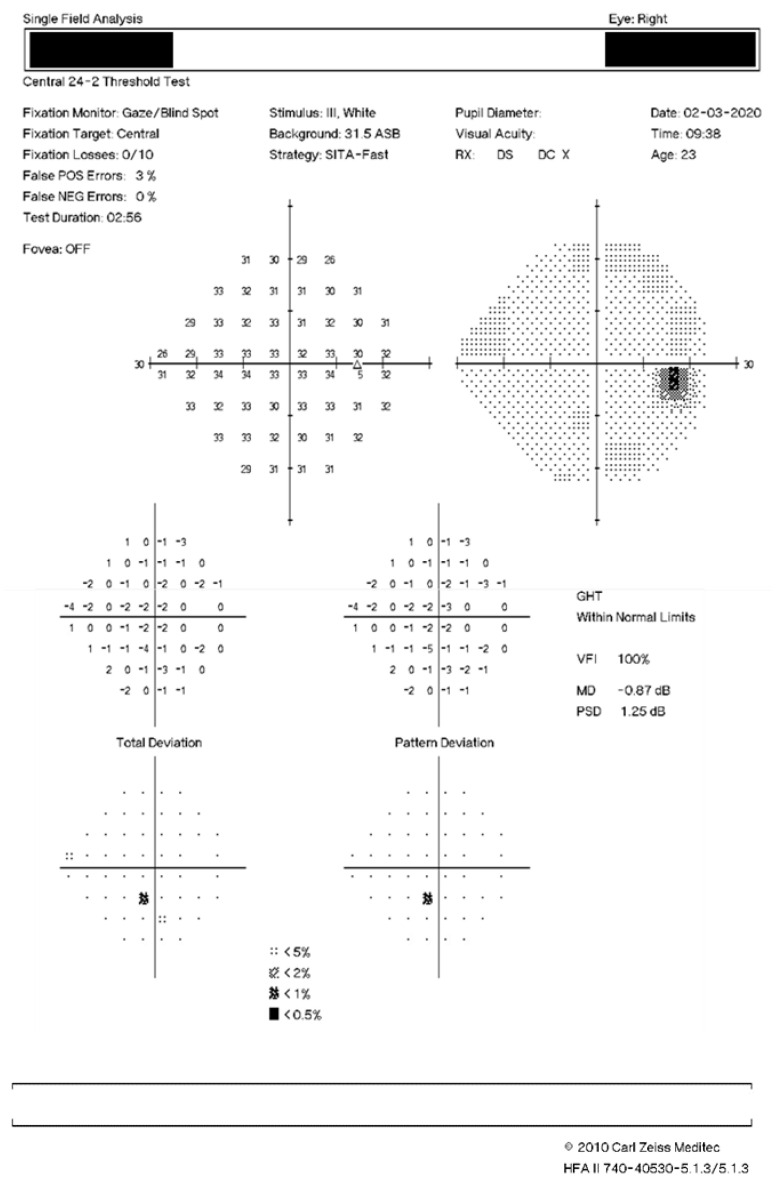

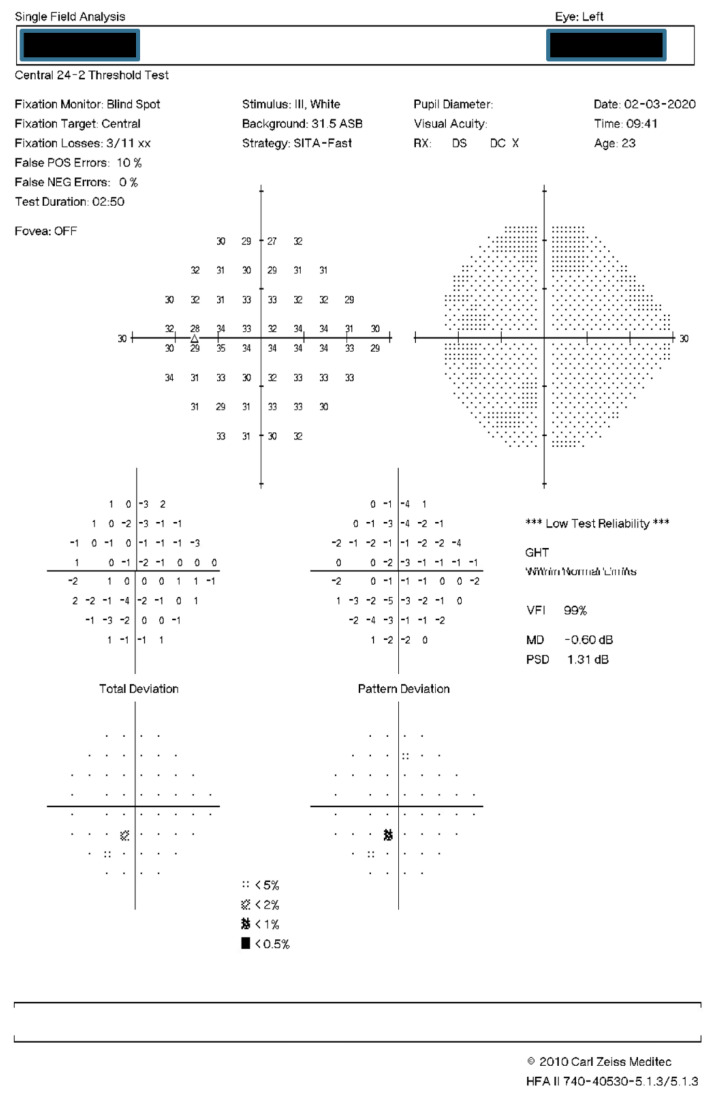
Visual field analysis March 2020. 24-2 Humphrey Visual Field of the right and left eyes taken one week after initial presentation showing no significant perimetry defects.

At initial Ophthalmology review, in February 2020, best-corrected visual acuity was 6/4 in the right eye and 6/5 in the left eye. Anterior segment examination was normal with briskly reactive pupils and no relative afferent pupillary defect, normal colour vision (Ishihara plates) and intraocular pressures. Optic discs were healthy with normal documented dilated fundus examination in both eyes. A spectral-domain optical coherence tomography (SD-OCT) scan of the macula was performed, which was initially erroneously interpreted as normal (Figure 1A,B). A review was planned for a week later, at which time the patient was seen by the same doctor. Ophthalmic examination remained unchanged, and a formal (Humphrey 24-2) visual field assessment was performed; no significant defects were noted, despite the persistent negative scotomata reported (Figure 2). Her symptoms were felt to be most likely a migrainous phenomenon, and she was discharged from Ophthalmology at this time. Her symptoms of bilateral discrete negative scotomata persisted despite the headache resolving, and her General Practitioner arranged an MRI brain scan the following month, the result of which was normal, and referred the patient for a Neurology opinion.

**Figure 3:**
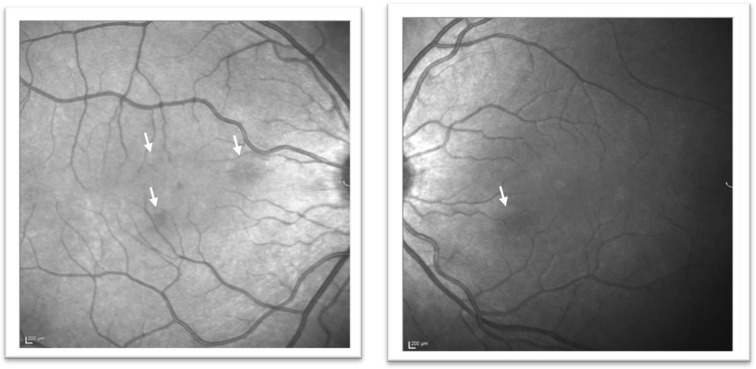
Near-infrared reflectance images in December 2020. The lesions seen on near-infrared reflectance imaging from initial presentation are seen to have become much less prominent over the 10-month period (white arrows).

A neurologist (JAC) reviewed the patient in July 2020, diagnosed possible persistent negative visual aura in light of the above, and attempted treatment with lamotrigine which was unsuccessful. After further review she was then referred directly to an Ophthalmic retinal specialist (PJB) in December 2020 when she reported that the scotomata had remained static in her visual field over the preceding 10 months. This remained an isolated symptom, and she had experienced no further headache episodes. On examination at this time (December 2020), her best-corrected visual acuity was 6/6 in the right eye and 6/5 in the left eye. She was orthophoric, and optic nerve function, including colour vision, pupils, and confrontational visual fields were normal. Anterior segment examination and intraocular pressures remained normal in both eyes. Dilated examination confirmed clear ocular media, no intraocular inflammation, and bilateral healthy optic discs. However, subtle red-brown perifoveal macular changes were noted in both eyes, with three lesions in the right eye and one in the left. A repeat macular SD-OCT scan with infrared-reflectance image was performed (Figure 1B and Figure 3) and the previous images were reviewed.

Despite being overlooked at the time, the near-infrared reflectance image from February 2020 did, in fact, demonstrate three discrete perifoveal hyporeflective lesions in the right eye and one in the left (Figure 1A), with SD-OCT scans demonstrating hyperreflective bands in the outer nuclear (ONL) and outer plexiform layer (OPL) and additional loss of clarity of the outer retinal bands (Figure 1B). These lesions corresponded to the lesions seen on clinical examination in December, and to the negative scotomata the patient had been reporting. The lesions highlighted on near-infrared reflectance had become significantly less prominent over the preceding 10 months (Figure 3). Based on the clinical findings, and in keeping with the history and patient demographics, a diagnosis of acute macular neuroretinopathy was made. The patient was reviewed again in March 2021, at which time symptoms persisted but were less prominent. The lesions seen on near-infrared reflectance were continuing to resolve, but ONL/OPL hyperreflectivity persisted on SD-OCT although this was progressively less prominent on the December 2020 and March 2021 scans, with some improvement also seen in the outer retinal band configuration (Figure 1B). 

## 3. Discussion

Acute macular neuroretinopathy is a rare disorder that typically affects young, white, female patients. The most common presentation is of scotomata, which tend to be paracentral, generally sparing fixation, and preserving visual acuity [2]. Pathogenesis of the condition is complex, and remains poorly understood, but a study of the retinal vasculature using optical coherence tomography angiography suggests an underlying compromise in the inner choroidal and deep retinal capillary circulations [6]. In keeping with this, many of the known associated risk factors, including oral contraceptive use [1], sympathomimetic exposure [7], systemic shock [8], and caffeine consumption [9], can be seen as vasoactive events.

Typically, lesions correspond to the reported scotomata, and are described as wedge-shaped, reddish-brown in colour, often arranged in a petaloid pattern, and are present in the perifoveal region of the retina with the apices pointing towards the fovea [1]. However, these lesions are often clinically subtle and sometimes not clinically visible at all, with some reports of a lag between symptomatic onset and appearance of visible lesions [10]. As in this case, although initially overlooked, near-infrared reflectance imaging may helpfully reveal the well-defined lesions responsible for the scotomata in the absence of clinical changes [2]. Our case highlights the importance of a detailed fundal examination, complete with scrutiny of ancillary imaging, to help differentiate a retinal cause for visual symptoms in the context of symptom overlap with migraine.

Visual field defects on formal testing are common in AMN. Although in our case perimetry was found to be normal (Figure 2), the resolution of the test performed may have been inadequate to identify any defect.

SD-OCT is a useful ancillary test, showing the early and characteristic feature of hyperreflectivity in the outer nuclear and outer plexiform layers, as demonstrated in our case, which resolves with time [4]. In the long-term, focal outer nuclear layer thinning typically occurs, representing photoreceptor compromise [4]. Recovery in AMN is variable, with reports ranging from resolution of symptoms in three days [10] to persistence of scotomata at nine years [11]. Persistence of outer nuclear layer atrophy seen on SD-OCT may represent long-term neural loss [4], which is consistent with adaptive optics imaging showing cone photoreceptor disruption with incomplete recovery in some cases [12], and reports of persistent abnormalities seen on multifocal electroretinography [13,14]. Long-term outer nuclear layer thinning may be a prognostic indicator, seemingly corresponding to persistence of symptoms and perimetry defects [4].

Migraine shares some of the trigger factors also associated with AMN, including caffeine use [15]; pharmacological agents, especially nitroglycerin [16]; sympathomimetic exposure; and hormone imbalance, including oral contraceptive use [17]. In migraines, negative scotomata are a well-described and common form of visual aura, however typical aura symptoms are defined as lasting no longer than one hour [18]. Whilst persistent aura without infarction is a recognised entity [18], this diagnosis—where aura symptoms otherwise typical of the patient’s previous auras last a week or more, in the absence of neuroradiological evidence of infarction—is extremely rare, with only around 40 cases reported in the literature, and most of these did not have SD-OCT performed [19]. Furthermore, a review on the subject of persistent visual aura symptoms in the context of migraines does not mention OCT, or the ophthalmological examination, at all [20].

OCT is a technique that requires knowledge and experience; as with any test, the experience of the examiner varies. In clinical reality, a neurologist, when told by an ophthalmologist that no other abnormality is found, will not immediately challenge this; that is why this case report is important, both for neurologists to challenge the existence of the concept of persistent negative visual aura and for ophthalmologists to raise awareness of AMN and the use (and earlier review) of OCT in this context.

## 4. Conclusions

This case is a reminder that caution should be taken in assigning a clinical presentation to migraine where symptoms deviate from the patient’s usual symptoms, or where suspected visual aura persists for days. In these cases, due care should be taken to evaluate for other causal diseases. Clinicians should be mindful of the possibility of acute macular neuroretinopathy in patients presenting with uni- or binocular scotomata. Ancillary tests, such as near-infrared reflectance imaging, SD-OCT scans, and formal perimetry, may be diagnostically revealing, and must be closely examined for abnormalities, even in the context of an initially normal fundus examination. Given that ophthalmological examination including OCT appears not to be routinely performed, even in cases of presumed persistent migraine visual aura, it is likely that misdiagnosis has rarely been contemplated. It is therefore hoped that this case report will trigger clinicians to consider this.

## Data Availability

Data is contained within the article.
